# Should the findings of the TASTE and TOTAL trials change clinical practice?

**Published:** 2016

The TOTAL and TASTE trials were undertaken to evaluate whether mechanical thrombus aspiration should routinely accompany primary percutaneous coronary intervention (PCI) for STEMI.

Evaluating the evidence prior to the two trials, Dr David Kettles from East London, South Africa, observed that it’s been known for a long time that thrombus is the enemy of good cath lab outcomes. ‘In STEMI, epicardial flow does not equal reperfusion. Distal embolisation is often a problem and angioplasty and stenting can contribute to this. Fifteen per cent of patients undergoing primary PCI have visible distal emboli, and myocardial perfusion after primary PCI is the strongest predictor of mortality.’ He was speaking at AfricaPCR 2016.

Aspiration is one of multiple approaches to deal with distal embolisation of thrombus and atherosclerotic debris. Many studies have suggested that manual thrombus aspiration improves ST-segment resolution and various surrogate markers.

‘It’s pathophysiologically plausible and relatively simple. It makes PCI easier and may have short- and long-term benefits. But what about the risk of complications? While the literature tends to downplay these, there is very possibly a higher stroke risk in real-world settings. It’s possible the trials prior to TASTE and TOTAL underestimated the risks. So we needed these randomised, controlled trials powered for mortality.’

Reviewing the two trials, Dr Hellmuth Weich, from Cape Town, South Africa, observed that TASTE was a multicentre, randomised, controlled trial evaluating all-cause mortality at 30 days in patients undergoing primary or rescue PCI for STEMI, either with or without thrombus aspiration. Patients were randomised after angioplasty.

There was virtually no difference in outcomes between the two arms at 30 days (2.8 vs 3.0%), with comparable findings at one year. ‘While it was a good trial, it may have been underpowered. There was very low mortality overall and there might possibly have been a selection bias, given that patients were randomised post angioplasty.’

TOTAL was a bigger trial than TASTE, and subjects were randomised prior to angioplasty. Its primary endpoint was a composite of cardiovascular death, recurrent myocardial infarction, class IV heart failure and cardiogenic shock at six months.

Once again, there was no significant difference in outcomes between the two arms of the trial. Of concern, however, was an increase in stroke in the aspirated arm that continued up to six months after the procedure.

Dr Weich noted in conclusion that there is therefore no evidence to suggest that manual thrombus aspiration be undertaken routinely. While the trials were interesting, it should also be kept in mind that the patient populations were not the same as those in Africa.

In the discussions that followed, the feeling was that the trials don’t tell interventional cardiologists not to undertake aspiration, just not to do it routinely. Patient selection is therefore an important concern, as is technique, in order to prevent stroke. ‘Pay careful attention to the guiding catheter and start aspiration 2 cm proximal to the lesion’, said Dr Kettles. ‘Employ multiple slow-passage techniques – at least two or three passes. Withdraw the aspirate catheter under aspiration and aspirate the guide thereafter.’ Another important determinant is the size and extent of the thrombus.

Summing up the key learnings, Dr William Wijns, chairman of PCR, made the following points:

There is no evidence of benefit of systematic mechanical aspiration during PCI based on primary or secondary efficacy measures.
There’s a possible safety signal in the form of increased stroke rates; this is a hypothesis-generating finding.
Operators may still choose thrombus aspiration in individual cases in order to facilitate procedural technique, bearing in mind that it will have no benefit in respect of endpoints such as mortality

‘There is an open door for its selective use, for example in cases of significant thrombus burden. Unmet needs remain, and we require better tools to remove thrombus efficaciously while protecting the myocardium. We also need to bear in mind that TOTAL and TASTE are not fully relevant to Africa, where the dominant treatment of STEMI is pharmaco-invasive and most patients are late presenters.’

